# A Population‐Wide Exploration of the *THAP11* CAG Repeat Size and Structure in the 100,000 Genomes Project and UK Biobank

**DOI:** 10.1002/mds.30073

**Published:** 2024-12-09

**Authors:** Chris Clarkson, Zhongbo Chen, Clarissa Rocca, Bharati Jadhav, Kristina Ibañez, Mina Ryten, Andrew J. Sharp, Henry Houlden, Arianna Tucci

**Affiliations:** ^1^ Clinical Pharmacology and Precision Medicine, William Harvey Research Institute, School of Medicine and Dentistry Queen Mary University of London London UK; ^2^ Department of Neuromuscular Diseases UCL Queen Square Institute of Neurology London UK; ^3^ Department of Clinical and Movement Neuroscience UCL Queen Square Institute of Neurology London UK; ^4^ The Francis Crick Institute London UK; ^5^ Department of Genetics and Genomic Sciences and Mindich Child Health and Development Institute Icahn School of Medicine at Mount Sinai New York New York USA; ^6^ UK Dementia Research Institute at Cambridge University of Cambridge Cambridge UK; ^7^ Department of Neurodegenerative Disease UCL Queen Square Institute of Neurology London UK

## Abstract

**Background:**

A CAG repeat expansion in *THAP11* was recently found to be associated with spinocerebellar ataxia in two Chinese families. Expanded repeats ranged from 45 to 100 units, with CAA sequence interruptions in the 5′ region and an uninterrupted CAG tract in the 3′ tail.

**Objective:**

Here, we assess the population distribution of the *THAP11* repeat, and its contribution to neurological diseases.

**Methods:**

We interrogated data from 54,788 individuals from Genomics England, 10,686 patients from the UCL Queen Square Institute of Neurology in‐house database (UCL IoN), and 424,340 individuals from the UK Biobank.

**Results:**

We identified expanded repeats in four individuals with learning difficulties without ataxia and in three individuals in UK Biobank, one with hereditary ataxia, one with hereditary neuropathy, and one with neurodegenerative disease. We showed a linear relationship between the number of CAA interruptions and overall repeat length.

**Conclusions:**

These results indicate that *THAP11* expansions are rare in the British population and that sequence structures predisposed to expansions may be more common in non‐British ancestries. © 2024 The Author(s). *Movement Disorders* published by Wiley Periodicals LLC on behalf of International Parkinson and Movement Disorder Society.

## 1 Introduction

Spinocerebellar ataxias (SCAs) are a diverse group of autosomal dominant neurodegenerative disorders characterized by progressive cerebellar dysfunction and incoordination.^1^ Despite significant strides in elucidating the genetic underpinnings of SCAs, a substantial proportion of cases remain unexplained, necessitating ongoing exploration for novel causative mutations and their pathomechanisms.^1^, ^2^ Tan et al. discovered an exonic CAG repeat expansion in the *THAP11* gene associated with late‐onset ataxia in two Han Chinese families.^3^ The pathogenic repeat size ranged from 45 to 100 triplets. Within the expanded repeat tract, CAA sequence interruptions were observed, with the uninterrupted CAG expansion (32–87 repeat units) occurring at the 3′ tail in affected individuals in the studied families. Fearnley et al. elaborated this work by finding the first European individual presenting with ataxia with a repeat size within the pathogenic range. Note that in this individual an expanded pathogenic allele was also found in *CACNA1A*, the repeat that causes SCA6.^4^ More recently, Hsiao and colleagues did not find any *THAP11* repeat expansions in a cohort of individuals with ataxia from Taiwan.^5^

To build on these findings, we examined the distribution of *THAP11* repeat alleles in individuals with and without neurological disease, across diverse ancestries using the Genomics England dataset and our in‐house exome database (UCL Queen Square Institute of Neurology, UCL IoN).^6^ We aimed to ascertain the prevalence and size of *THAP11* expansions beyond the initially identified Chinese cohorts. We also analyzed the diversity of the sequence structures in the *THAP11* locus to assess population differences. Finally, we studied phenotypic associations of *THAP11* expansions in the UK Biobank.

## 2 Methods

We characterized the number of repeats at the *THAP11* repeat locus (GRCh38 position chr16: 67,842, 863‐67, 842,950) using sequencing data from two different cohorts:

- Genomics England
  - 50,233 individuals from the 100KGP cohort (WGS data), comprising 14,186 participants with a known neurological disorder (803 of whom have ataxia and 6368 were intellectual disability cases), and 36,047 controls presenting with non‐neurological disorders;
  - 4555 individuals from the Genomics Medicine Service (GMS) in the UK (whole‐genome sequencing [WGS] data), comprising 3929 patients with a neurological disorder (287 of whom have ataxia and 1447 were intellectual disability cases) and 626 controls who do not have a neurological disorder.
- Our in‐house exome database (UCL Queen Square Institute of Neurology, UCL IoN): 10,686 individuals and family members with whole exome sequencing data with suspected neurogenetic disease from our in‐house UCL IoN database, including 887 individuals with ataxia, and 1977 with intellectual disability.

To estimate *THAP11* repeat sizes, we used ExpansionHunter (version 5).^7^ Genetically predicted ancestries were supplied with the data from the 100KGP project cohort for the 36,047 controls. This population was used to summarize the spread of repeat sizes across different genetic ancestries.

We then visualized the read data in individuals with repeat sizes ≥45 to review the accuracy of these ExpansionHunter calls manually. Calls were required to have ≥2 spanning reads and ≥10 flanking reads that concurred with the spanning read(s) to be considered genuine. This was done using REViewer.^8^

Finally, we used our custom package, Repeat Crawler (https://github.com/chrisclarkson/gel/), developed to characterize the structure of the *THAP11* repeat sequence. Repeat Crawler works by taking the read data from curated binary alignment map (BAM) files and reporting the presence or absence of different motifs in accordance with a pre‐ordered set of repeat components. A Wilcoxon rank sum test was performed to compare the difference between disease cohorts and controls. A linear model was used to describe the relationship between the number of repeat interruptions and overall repeat length. These analyses were performed using Python (version 3.7) and R (version 4.02).

From the set of UK Biobank (UKB) individuals with Illumina 150 base pair (bp) paired‐end WGS data, we partitioned these into major ancestral groups using an approach based on principal component analysis of a set of ~64,000 high‐quality linkage disequilibrium pruned single nucleotide variants (SNVs) with minor allele frequency >5%, as described previously.^9^ Code used for ancestry assignment is available at https://zenodo.org/doi/10.5281/zenodo.10820994. Using pairwise kinship coefficients provided by the UKB, for samples with second‐degree relationships or higher (kinship coefficient >0.0883), we randomly retained only a single unrelated individual. We also removed individuals with predicted sex chromosome aneuploidy based on read depth analysis of the sex chromosomes and removed individuals who we were notified had withdrawn their consent for inclusion in the UKB study. After these filtering steps, we retained data for 424,340 unrelated individuals for association analysis. Of these individuals, 411,265 were European, 6455 were African, 3822 were South Asian, 1587 were East Asian, and 1211 were American.

Using the UKB Research Analysis Platform, we performed genotyping of the *THAP11* repeat with ExpansionHunter (version 5). From the resulting diploid genotypes output by ExpansionHunter, we utilized data only for the longer of the two alleles per sample at each locus, discarding data from the shorter allele. We considered individuals carrying *THAP11* repeat alleles ≥45 copies as having putative expansions.

We utilized phenotype data for individuals in the UKB derived from a selected set of 133 quantitative, categorical, and binary traits related to educational attainment, cognitive function, and neurological disease, accessed through UKB application number 82094 and processed as described previously.^10^ Associations of *THAP11* expansions with each selected trait were performed using linear regression for quantitative traits or logistic regression for binary traits in the brglm R package (v 0.7.2), incorporating covariates of ancestry, sex, age, WGS insert size, sequencing center, and the top five principal components derived from analysis of SNVs to account for ancestry. We applied a Bonferroni correction for multiple testing based on the 133 traits analyzed. Full results are shown in Supplementary Tables S2 and S3.

## 3 Results

**FIG. 1:**
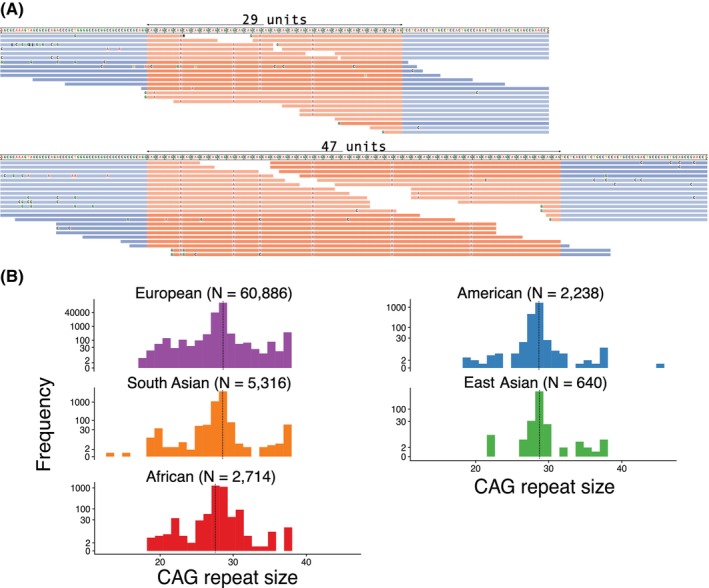
(A) Exemplary pileup plot from an individual with a predicted repeat size of 47 repeat units. (B) The spread of repeat sizes found across our control cohort (n = 36,047). The cohort is divided and colored by genetic ancestry and the number of alleles is noted for each. The median value of each distribution is marked with a dotted line (28 in Africans and 29 for all others). [Color figure can be viewed at wileyonlinelibrary.com]

We initially studied a total of 54,788 individuals with WGS data, comprised of a set of 18,115 patients with neurological disease (n = 14,186 from 100KGP and n = 3929 from GMS) and 36,673 controls without neurological symptoms (n = 36,047 from 100KGP and n = 626 from GMS). In the neurological cohort, none of the patients with ataxia carried *THAP11* alleles with ≥38 repeats. However, we identified three people with *THAP11* alleles containing ≥45 repeats: two with intellectual disability, of European genetic ancestry (47 repeats), and one control of American genetic ancestry (45 repeats). We also analyzed our in‐house exome sequencing database comprising data from 10,686 people with suspected inherited neurological diseases (UCL Koios database, including 1977 with intellectual disability), and identified two additional individuals with intellectual disability and expanded *THAP11* repeats (47 repeats). The genotypes were inspected manually (Figs. 1A and S1). The clinical data for all individuals with the repeat expansion were reviewed. While two individuals from the 100KGP cohort were found to have non‐syndromic intellectual disability with autism, the remaining had similar Human Phenotype Ontology (HPO) terms characterized by developmental delay and microcephaly, and none were diagnosed with ataxia. Furthermore, the history and genotypes of the families of each of the individuals were assessed. For one of these individuals, their unaffected father was found to carry the same expanded allele as their affected daughter. For all others, no parental data was available (Table S1). In all individuals with ≥45 repeats, the expanded tract was interrupted by 6 CAA sequences, and the 3′ uninterrupted CAG tract was either 9 or 10 repeats (Fig. S1).

We harnessed the UKB to further evaluate the potential association of *THAP11* expansions with neurological disorders. Using data from >400,000 UKB participants, we did not find any evidence of association of *THAP11* expansions (≥45 repeats) with lower educational attainment or reduced cognitive function. However, we identified significant associations with several rare neurological disorders, although each of these were based on just a single individual (Tables S2 and S3). Three individuals with neurological disorders carried the *THAP11* expansion: one with ataxia (45 repeats, also previously reported in Fearnley et al.^4^), one with hereditary motor and sensory neuropathy (46 repeats), and one with unspecified degenerative disease of the nervous system (47 repeats). Inspection of the read pileup plots showed that the largest 3′ uninterrupted tract of CAG motifs in these individuals was nine (Fig. S1).

Based on the fact that the original expansion was found in a Chinese family, we used our 100KGP control cohort to study the distribution of repeat sizes across genetic ancestries: 30,443 Europeans, 2658 South Asians, 1507 African, 1119 American, and 320 East Asian (Fig. 1B). No individual of East Asian genetic ancestry was found to have a repeat size ≥45, although this is likely due to the fact that only 320 individuals were tested.

**FIG. 2:**
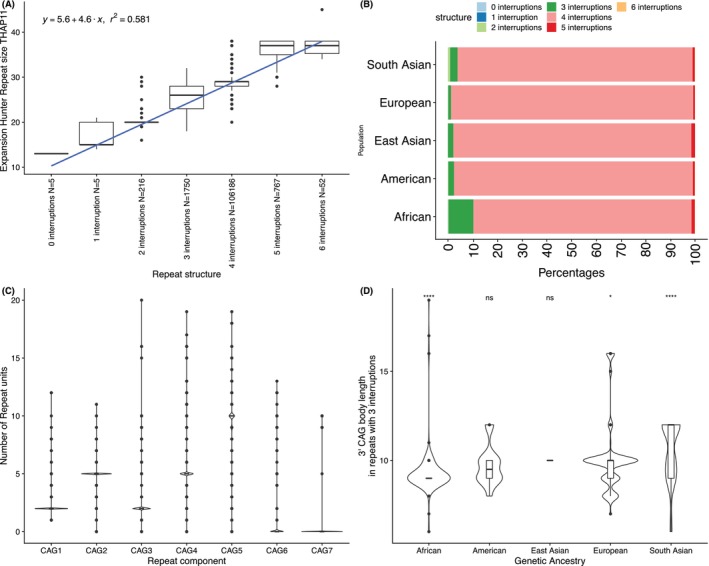
(A) *THAP11* sequence structure in the control cohort. The number of CAA interruptions in the CAG tandem stretch is plotted against the overall repeat length estimated by ExpansionHunter. (B) Bar plots document the prevalence of each repeat structure as a percentage (x‐axis) of all alleles in different genetic ancestries (y‐axis). (C) The number of repeat units in the CAG stretches between CAA interruptions is plotted on the y‐axis for each CAG component, numbered 1–7. (D) The spread of the repeat size of the 3' CAG repeat is documented (y‐axis) in alleles with only three CAA interruptions across different genetic ancestries (x‐axis). [Color figure can be viewed at wileyonlinelibrary.com]

Given that the length of the 3′ uninterrupted CAG tract within the *THAP11* repeat region has been proposed to drive the pathogenicity of the expansion, we characterized the structure of this locus. We documented the presence and length of the different components of the *THAP11* repeat locus across populations. Figure 2 (A) shows that the total length of the tandem repeat increases proportionally (*R* ^2^ = 0.58 and *P* < 2.2 x 10^−16^) to the number of CAA interruptions in the repeat body. Furthermore, we showed that the prevalence of alleles with three CAA interruptions (characteristic of the disease‐causing alleles) is most common in African and South Asian genetic ancestries, and that the length of the CAG repeats at the 3′ end of the tandem repeats with three CAA interruptions differs significantly in genomes of African and South Asian genetic ancestries from all others (Fig. 2).

## 4 Discussion

Tan et al. and Fearnley et al. postulated that CAG toxicity could be the driving factor in the pathogenicity of an expansion.^3^, ^4^ This was on the basis that Tan et al. observed fewer than four CAA interruptions and an expanded pure CAG repeat, at the 3′ end of the locus (longer than 31) in people with ataxia. In this study, we showed an enrichment for intellectual disability in people with >45 repeats with 6 CAA interruptions, and an uninterrupted 3′ tract of 9 or 10 CAG units. Subsequently, we utilized the UKB to show a potential association of repeat expansion with several neurological disorders including ataxia. The alleles driving this signal had uninterrupted CAG tracts no longer than nine repeat units. Hence, no expanded repeat in the individuals studied here had a large uninterrupted CAG expansion. Despite having relatively short pure CAG tracts, the larger repeat sizes being enriched in intellectual disability and neurological disease cases could be due to the disruption of a pathway that is different to that described in Tan et al.^3^ Indeed, Quintana et al. reported an association of *THAP11* mutants with developmental disorders by disrupting cobalamin metabolism, despite the fact that the concerned mutations are in different parts of the gene.^11^

To describe the distribution of alleles in the general population, we further explored the sequence structure of the *THAP11* locus. We showed that the total number of repeats in the locus increases in proportion to the number of CAA interruptions. The mechanism that accounts for the association between CAA interruptions and CAG repeat size might be unequal allelic homologous recombination leading to duplication and contractions of the repeated tract (including the CAA interruption), a mechanism that has been proposed to occur and generate many of the described polyalanine expansions in oculopharyngeal muscular dystrophy.^12^ In this study, genomes with African and South Asian genetic ancestries had the highest prevalence of alleles with three CAA interruptions. Furthermore, the 3′ CAG repeats of these alleles were significantly different in these ancestries versus all others. This suggests that repeat structures susceptible to expansions may be more common in under‐sequenced populations, and expansions in *THAP11* will likely be rare or absent in European‐centric biobanks.

## Author Roles

(1) Research Project: A. Conception, B. Organization, C. Execution, D. Bioinformatics Analysis, E. Interpretation of Results; (2) Statistical Analysis: A. Design, B. Execution, C. Review and Critique; (3) Manuscript Preparation: A. Writing of the First Draft, B. Review and Critique.

C.C.: 1D, 1E, 3A, 3B.

Z.C.: 1D, 1E, 3A, 3B.

C.R.: 1D, 1E, 3A, 3B.

B.J.: 1D, 1E, 3A, 3B.

M.R.: 1E, 3A, 3B.

H.H.: 1E, 3A, 3B.

K.I.: 1E, 3A, 3B.

A.J.S.: 1E, 3A, 3B.

A.T.: 1A, 1E, 3A, 3B.

## Financial Disclosures

The authors declare that there are no additional disclosures to report.

## Ethical Compliance

Ethical approval for the 100,000 Genomes Project was granted by the East of England Cambridge South Research Ethics Committee (reference 14/EE/1112). Collection of the UK Biobank (UKB) data was approved by the Research Ethics Committee of the UKB obtained under application 32568. All study participants provided informed consent, and the protocols for UKB are overseen by The UK Biobank Ethics Advisory Committee (see https://www.ukbiobank.ac.uk/ethics/). All authors confirm that they have read the Journal's position on issues involved in ethical publication and affirm that this work is consistent with those guidelines.

## Supporting information


**Figure S1.** (A–H) REViewer was used to create pileup plots in the eight different individuals with predicted repeat sizes ≥45. Panels (A, B) show the intellectual disability cases from the 100KGP platform, while (C, D) are from the UCL Koios database, and (E–G) are from the UK Biobank individuals with neurological symptoms.

**Table S1.** Details of individuals with *THAP11* expansions from Genomics England and the UCL Queen Square Institute of Neurology in‐house exome database (UCL IoN).

**Table S2.** Documents results from logistic regression analysis performed on UK Biobank data.

**Table S3.** Documents results from quantitative trait analysis performed on UK Biobank data.

## Data Availability

The data that support the findings of this study are available from Genomics England, UCL and UK Biobank. Restrictions apply to the availability of these data, which were used under license for this study. Data are available from https://www.genomicsengland.co.uk/ with the permission of Genomics England, UCL and UK Biobank.
