# Supplementary material for: A Population‐Wide Exploration of the THAP11 CAG Repeat Size and Structure in the 100,000 Genomes Project and UK Biobank
Source: Mov Disord. 2024 Dec 9;40(3):561–6. doi: 10.1002/mds.30073 (PMC11926500; doi:10.1002/mds.30073)
Supplement: Supplementary file 1 — Figure S1. (A–H) REViewer was used to create pileup plots in the eight different individuals with predicted repeat sizes ≥45. Panels (A, B) show the intellectual disability cases from the 100KGP platform, while (C, D) are from the UCL Koios database, and (E–G) are from the UK Biobank individuals with neurological symptoms. [file MDS-40-561-s001.docx]

(A)


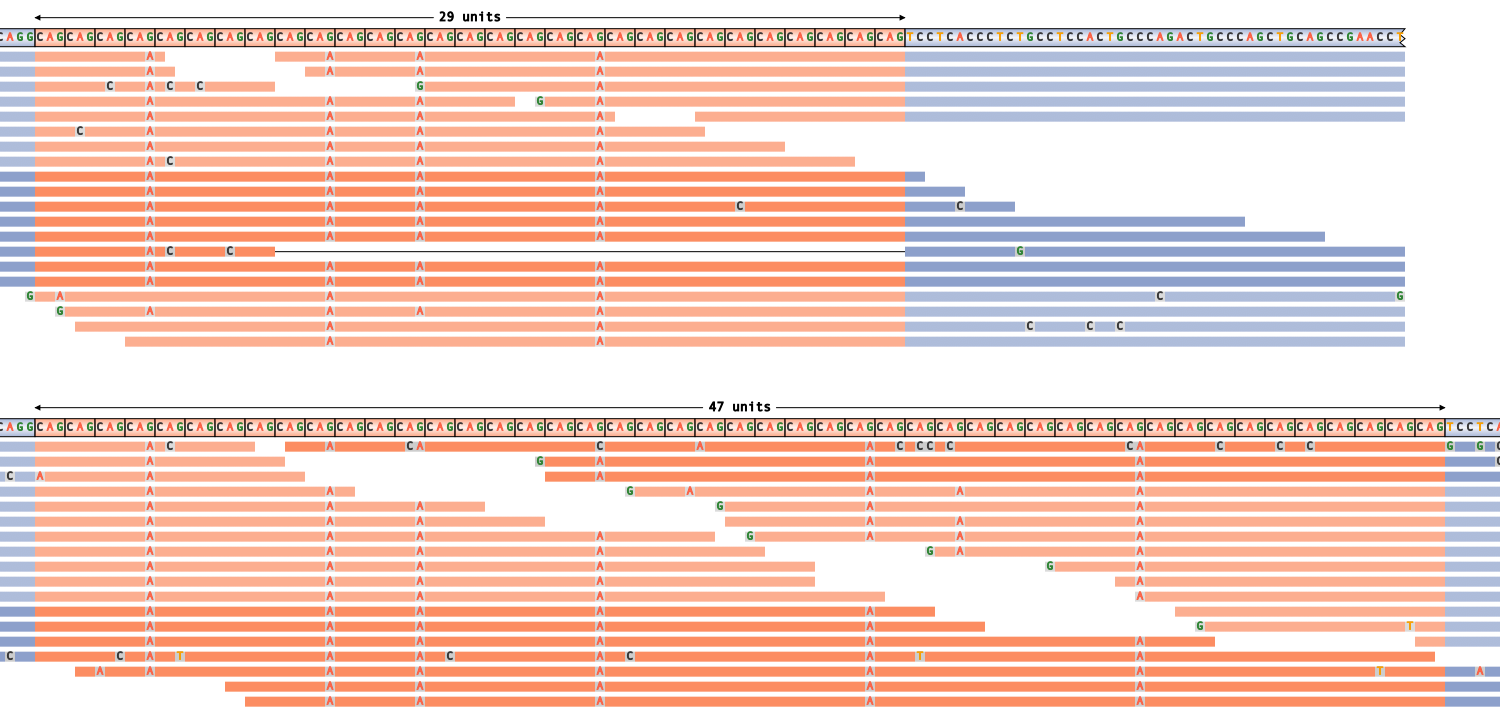


(B)


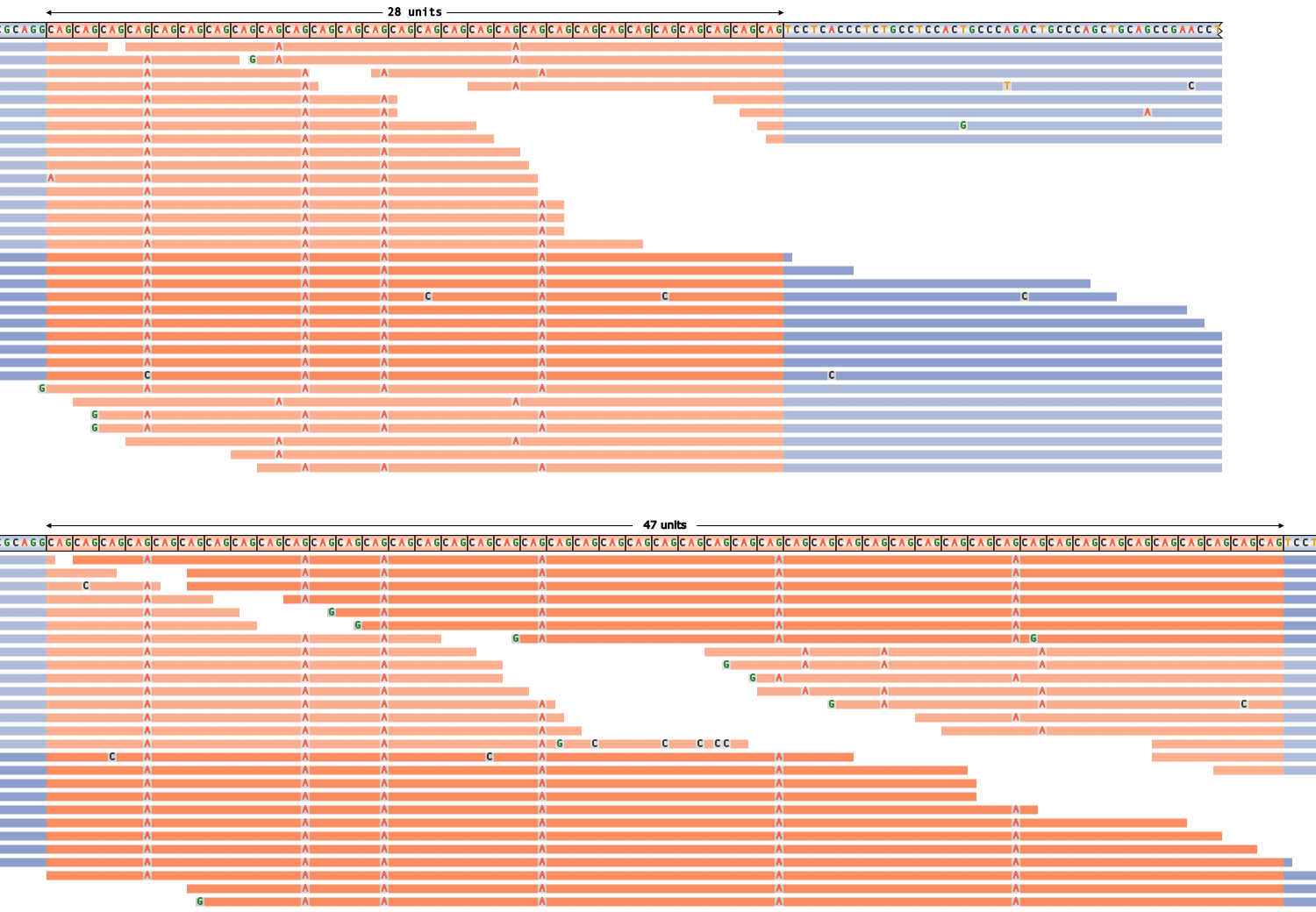


(C)


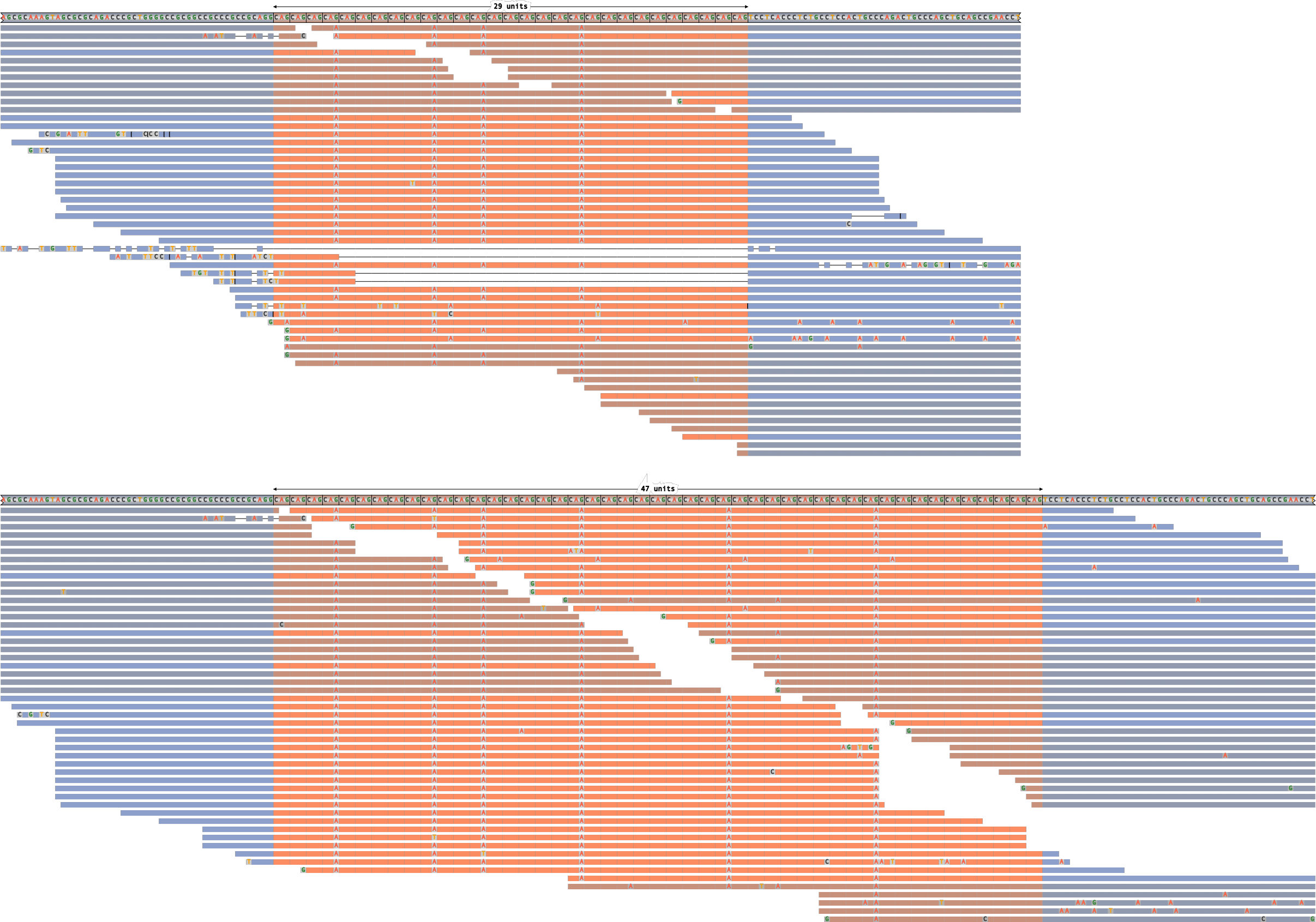


(D)
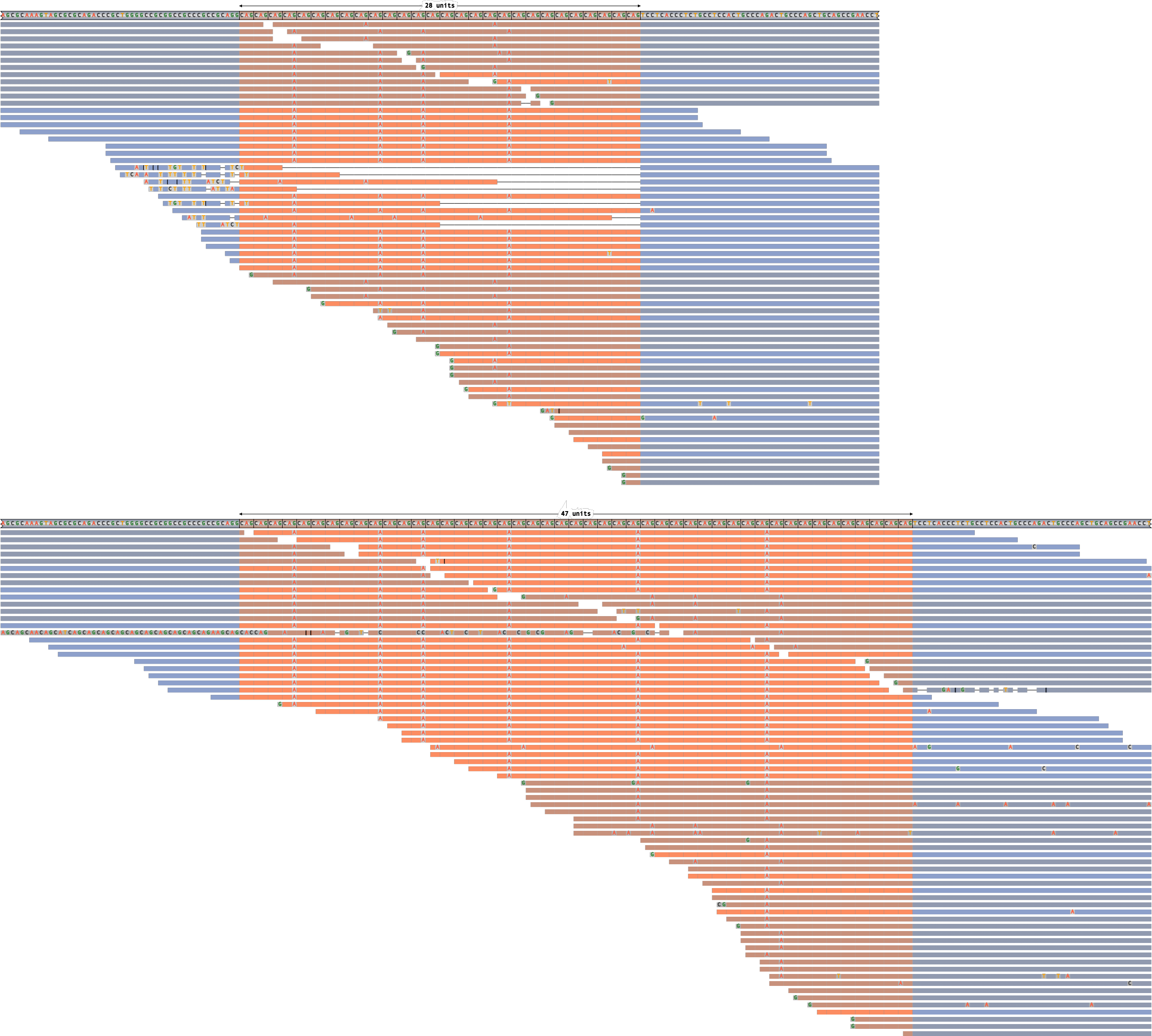


(E)


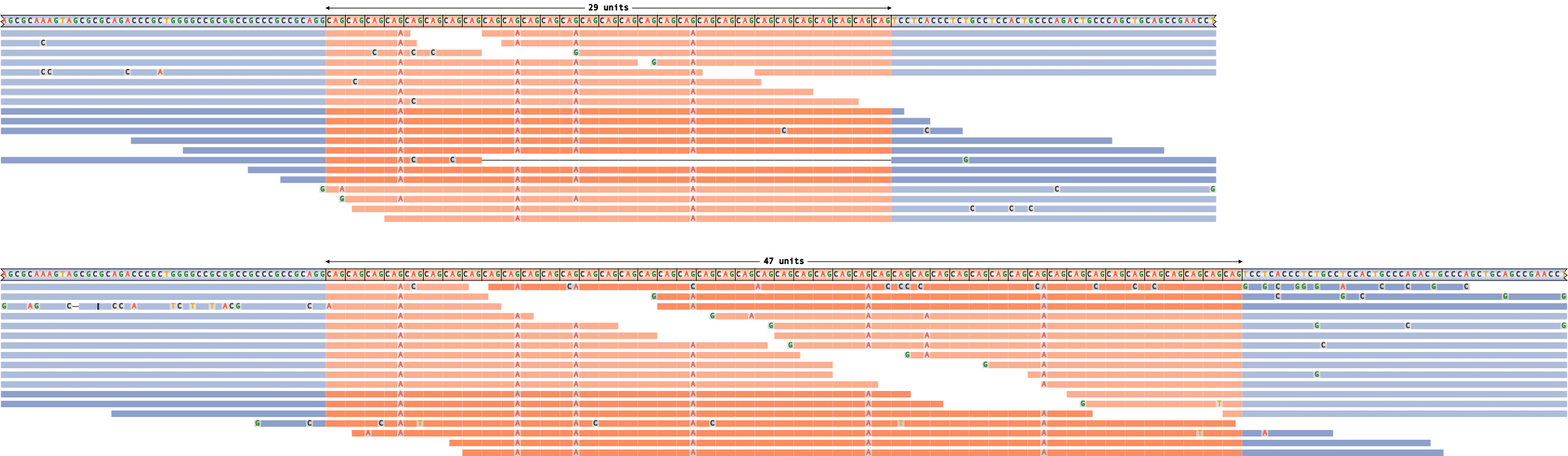


(F)


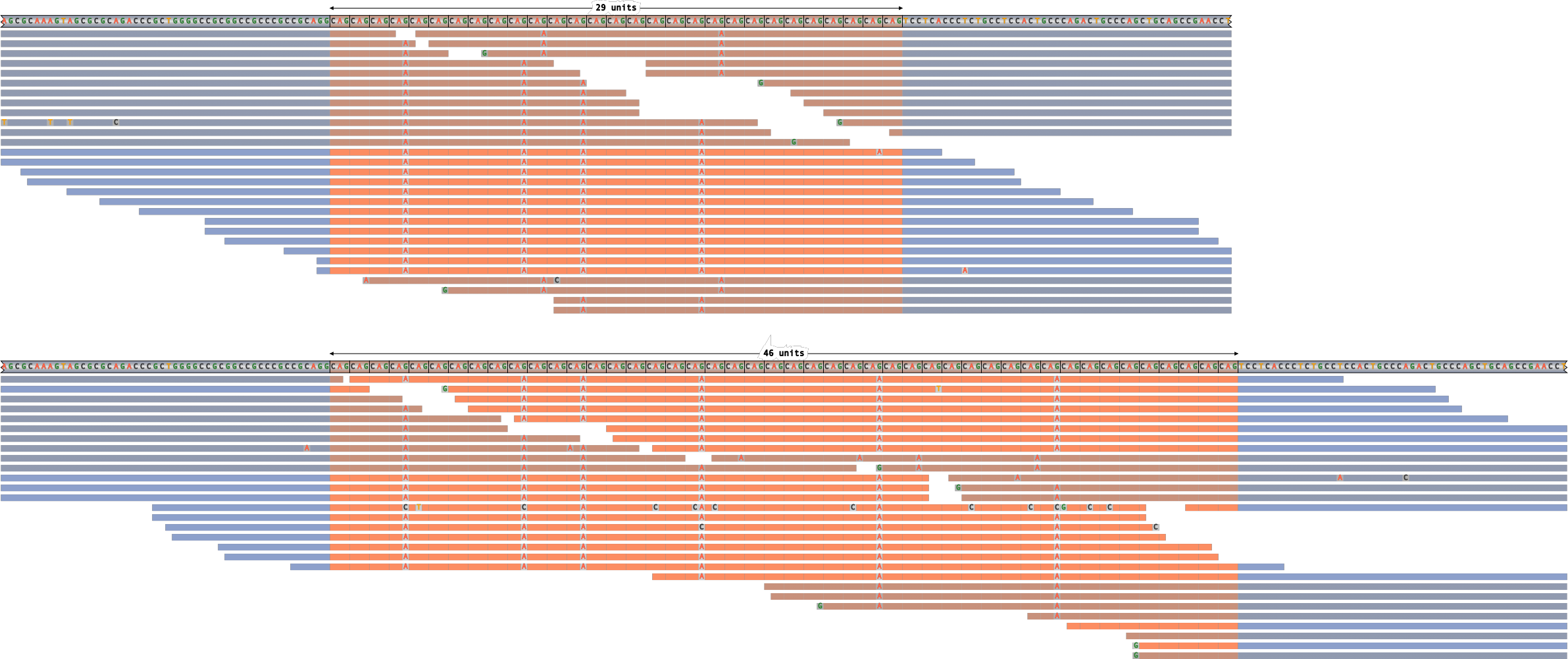


(G)


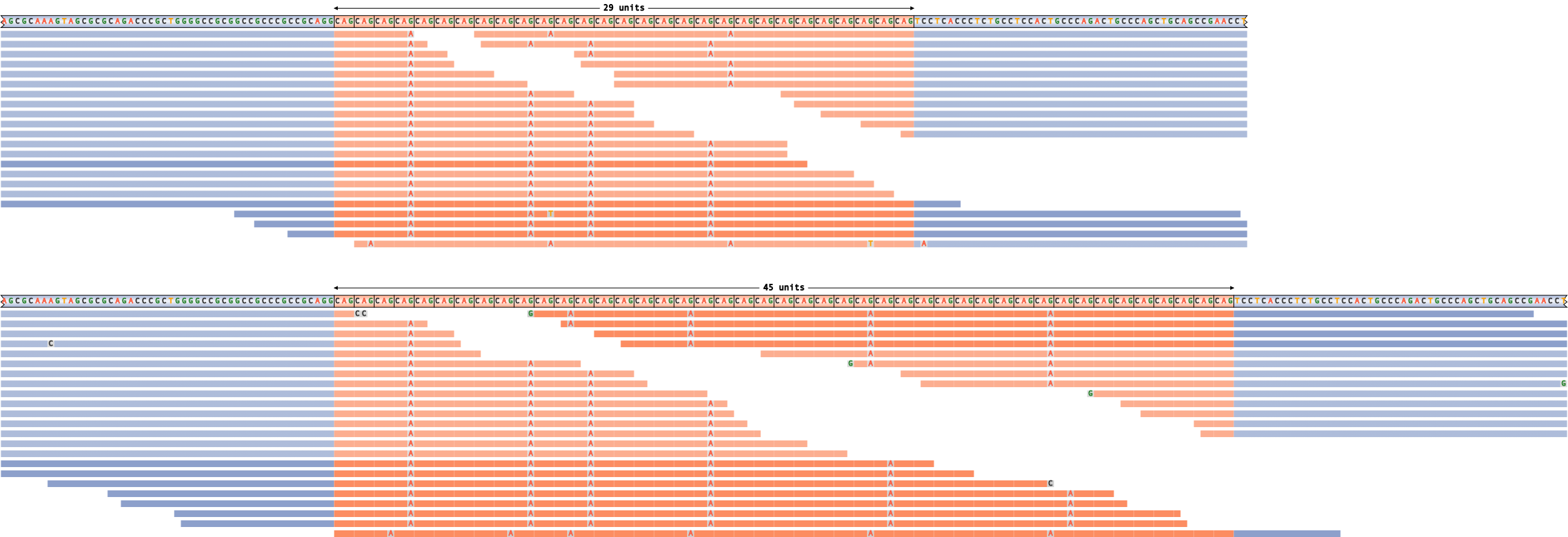


Supplementary figure 1: (A-H) REViewer was used to create pileup plots in the 8 different individuals with predicted repeat sizes ≥ 45. Panels (A-B) show the intellectual disability cases from the 100KGP platform, while (C-D) are from the UCL Koios database and (E-G) are from the UKBB individuals with neurological symptoms.
